# Public Health Implications of Cysticercosis Acquired in the United States

**DOI:** 10.3201/eid1701.101210

**Published:** 2011-01

**Authors:** Frank Sorvillo, Patricia Wilkins, Shira Shafir, Mark Eberhard

**Affiliations:** Author affiliations: University of California, Los Angeles, California, USA (F. Sorvillo, S. Shafir);; Touro University International, Cypress, California, USA (F. Sorvillo);; Los Angeles County Department of Public Health, Los Angeles (F. Sorvillo);; Centers for Disease Control and Prevention, Atlanta, Georgia, USA (P. Wilkins, M. Eberhard)

**Keywords:** Parasites, foodborne infections, cysticercosis, neurologic disease, Taenia solium, public health, epidemiology, tapeworms, United States, synopsis

## MEDSCAPE CME

Medscape, LLC is pleased to provide online continuing medical education (CME) for this journal article, allowing clinicians the opportunity to earn CME credit.

This activity has been planned and implemented in accordance with the Essential Areas and policies of the Accreditation Council for Continuing Medical Education through the joint sponsorship of Medscape, LLC and Emerging Infectious Diseases. Medscape, LLC is accredited by the ACCME to provide continuing medical education for physicians.

Medscape, LLC designates this Journal-based CME for a maximum of 1 *AMA PRA Category 1 Credit(s)*^TM^. Physicians should claim only the credit commensurate with their participation in the activity.

All other clinicians completing this activity will be issued a certificate of participation. To participate in this journal CME activity: (1) review the learning objectives and author disclosures; (2) study the education content; (3) take the post-test and/or complete the evaluation at www.medscapecme.com/journal/eid; (4) view/print certificate.

**Release date: December 22, 2010; Expiration date: December 22, 2011**

## Learning Objectives

Upon completion of this activity, participants will be able to:

- Describe the presentation and prevalence of cysticercosis acquired within the United States
- Describe how cysticercosis appears to be transmitted within the United States
- Describe public health strategies that could be useful in controlling cysticercosis transmission within the United States.

## Editor

**Nancy Mannikko, PhD, MS**, Technical Writer-Editor, *Emerging Infectious Diseases. Disclosure: Nancy Mannikko, PhD, MS, has disclosed no relevant financial relationships.*

## CME AUTHOR

**Laurie Barclay, MD, **freelance writer and reviewer, Medscape, LLC. *Disclosure: Laurie Barclay, MD, has disclosed no relevant financial relationships.*

## AUTHORS

*Disclosures:* ***Frank Sorvillo, PhD; Patricia Wilkins, PhD; Shira Shafir, PhD, MPH;*** *and* ***Mark Eberhard, PhD,*** *have disclosed no relevant financial relationships.*
