# Supplementary material for: Public Health Implications of Cysticercosis Acquired in the United States
Source: Emerg Infect Dis. 2011 Jan;17(1):1–6. doi: 10.3201/eid1701.101210 (PMC3298370; doi:10.3201/eid1701.101210)
Supplement: Related texts

# Review article: Public Health Implications of Cysticercosis Acquired in the United States

**DOI:** 10.3201/eid1701.101210

**Authors:** Frank Sorvillo, Patricia Wilkins, Shira Shafir, Mark Eberhard

**Affiliations:** Author affiliations: University of California, Los Angeles, California, USA (F. Sorvillo, S. Shafir);; Touro University International, Cypress, California, USA (F. Sorvillo);; Los Angeles County Department of Public Health, Los Angeles (F. Sorvillo);; Centers for Disease Control and Prevention, Atlanta, Georgia, USA (P. Wilkins, M. Eberhard)

## Abstract

Cysticercosis has emerged as a cause of severe neurologic disease in the United States that primarily affects immigrants from Latin America. Moreover, the relevance of cysticercosis as a public health problem has been highlighted by local transmission. We searched the biomedical literature for reports documenting cases of cysticercosis acquired in the United States. A total of 78 cases, principally neurocysticercosis, were reported from 12 states during 1954–2005. A confirmed or presumptive source of infection was identified among household members or close personal contacts of 16 (21%) case-patients. Several factors, including the severe, potentially fatal, nature of cysticercosis; its fecal–oral route of transmission; the considerable economic effect; the availability of a sensitive and specific serologic test for infection by adult *Taenia solium* tapeworms; and the demonstrated ability to find a probable source of infection among contacts, all provide a compelling rationale for implementation of public health control efforts.

Cysticercosis, infection with the larval stage of the pork tapeworm, *Taenia solium*, is a known cause of illness and death in humans (*1**,**2*). Neurocysticercosis, the most serious form of the disease, occurs when larvae invade the central nervous system. The disease is dependent on a 2-host life cycle in which humans serve as the definitive host and pigs as the intermediate host. Eggs, which are directly infectious, are shed in the feces of humans infected with the adult tapeworm. When these eggs are ingested by pigs, larvae emerge from the eggs, penetrate the intestinal mucosa, and disseminate through the bloodstream to various tissues where the larval stage, or cysticercus, develops. The cycle is completed when humans, the only naturally infected definitive host, consume raw or undercooked pork containing cysticerci, which attach to the small intestine and develop into the adult tapeworm. However, humans may also become infected with the larval stage when they ingest *T. solium* tapeworm eggs, typically in contaminated food or water. Cysticercosis is therefore a fecal–oral-transmitted disease acquired by ingestion of eggs excreted in the feces of a human tapeworm carrier.

Cysticercosis is widely prevalent in Latin America, Asia, and parts of Africa; ≈50 million persons are infected globally (*2**,**3*). The disease is routinely seen in the United States in immigrants from disease-endemic regions, particularly Latin America (*4**,**5*). Recent attention has focused on cysticercosis as one of the neglected infections associated with poverty in the United States and also as a major cause of preventable epilepsy (*6*). The relevance of this parasitic infection as a public health problem in the United States has been highlighted by reports of autochthonous cases (*7*). This phenomenon was underscored by Schantz et al. in their report of an outbreak of cysticercosis in an Orthodox Jewish community in New York City in 1990–1991 (*8*). Several other publications, particularly over the past 25 years, have documented cysticercosis acquired in the United States (*5**,**7**–**25*). We present an aggregation of these published reports and discuss the attendant public health issues, including policy implications and approaches to prevention.

A search of PubMed was conducted by using the terms cysticercosis, neurocysticercosis, *Taenia solium*, taeniasis, and United States. Web-based searches were also conducted by using the same terms. Articles were reviewed for cases of locally acquired cysticercosis or reference to other publications documenting cysticercosis in persons without a history of travel outside the United States. Chapters in books dealing exclusively with cysticercosis and selected infectious disease texts were also reviewed.

We defined a case of cysticercosis as confirmed if there were clinical manifestations consistent with cysticercosis and a positive serologic test result or evidence of infection based on biopsy findings. A presumptive case was defined by clinical and imaging studies indicative of cysticercosis but lacking serologic or biopsy confirmation. An autochthonous case was defined as cysticercosis in a US-born person with no history of travel to a disease-endemic area before onset of symptoms indicated in the publication. Case-patients with any such travel history, independent of duration, or those for which information on travel was not reported, were excluded from consideration as locally acquired.

Twenty publications from 1954 through 2004 that documented 78 total cases of US-acquired cysticercosis from 12 states were identified (Table 1). Five (25%) reports detailing 44 (56.4%) cases were from California, all from Los Angeles County. Neurocysticercosis was most commonly reported (97.4%), although ocular (*1*) and subcutaneous (*1*) infections were also observed. No deaths were reported but long-term follow-up of clinical status was rarely reported. Demographic information was typically not provided. However, among studies reporting such data, the mean age of case-patients with locally acquired infection was 21.3 years (range 14 months–80 years). Ages for several pediatric patients were not reported; therefore, the available data on age must be viewed with caution. Among those few reports providing information on gender, 10 cases were in female patients and 6 were in male patients. Information on race or ethnicity was rarely included.

**Table 1:** Published reports of cysticercosis cases that were acquired in the United States, 1954–2005

Location	Year of onset or report	No. cases	Form	Probable or suspected source	Reference
Minnesota	2005	1	Neurologic	NR	(*9*)
Chicago, IL	1996	2	Neurologic	NR	(*10*)
Oregon	1995–2000	5	Neurologic	1 had a household visitor from a disease- endemic area	(*11*)
Chicago, IL	1986–1994	8	Neurologic	4 had recent visitors from disease-endemic areas	(*12*)
Houston, TX	1985–1991	1	Neurologic	NR	(*13*)
South Carolina	1990	1	Neurologic	Neighbor’s friends from a disease-endemic area	(*14*)
Boston, MA	1990	1	Neurologic	Father with tapeworm	(*14*,*15*)
Los Angeles, CA	1988–1990	10	Neurologic	Household contact with tapeworm in 2 cases	(*16*)
New York	1989	4†	Neurologic	Domestic employees, 1 who had tapeworm	(*8*)
North Carolina	1989	1	Neurologic	Seasonal workers from disease-endemic area	(*14*)
Boston, MA	1986†	1	Neurologic	NR	(*17*)
Los Angeles, CA	1980–1986	14	Neurologic	NR	(*18*)
Denver, CO	1976–1985	1	Neurologic	NR	(*19*)
Los Angeles, CA	1981–1982	7	Neurologic	NR	(*20*)
Los Angeles, CA	1973–1983	12	Neurologic	NR	(*5*)
Los Angeles, CA	1979	1	Subcutaneous	Domestic employee who had tapeworm	(*21*)
Pennsylvania	1979†	1	Neurologic	Father who had tapeworm	(*22*)
Hershey, PA	1975	1	Ocular	NR	(*23*)
Louisiana	1954	3	1 case neurologic and subcutaneous	NR	(*24*)
Louisiana	1954	1	Neurologic	NR	(*25*)

*Some reports did not name specific cities within the state. NR, not reported. †There were 7 additional seropositive cases detected in the community from which the initial cases were identified.

Nine reports documented multiple cases of US-acquired cysticercosis (*5**,**7**,**9**–**11**,**15**,**17**,**19**,**23*). In the New York City cluster of neurocysticercosis, 4 initial cases in which patients experienced seizures were identified, and an additional 7 persons were found to be seropositive. Investigation determined that the probable sources of infection were domestic workers with tapeworm infection who had emigrated from areas endemic for taeniasis/cysticercosis.

In a pilot cysticercosis surveillance system conducted in Los Angeles County from 1988 through 1990, ten locally acquired cases were identified and represented 7% of 138 total incident cases (*15*). The mean age of these patients was 13.7 years (range 4–33 years), and 7 (70%) patients were female. Most patients (70%) were Hispanic, 2 (20%) were white, and 1 (10%) was black. It is noteworthy that 7 additional cases of cysticercosis were identified in immigrants from disease-endemic areas who were long-term residents (mean length of residency 15.4 years) and had no history of additional travel or exposure outside the United States since their immigration (Table 2). This phenomenon has also been recognized by McCormick (*20*), who reported 20 cases of cysticercosis among foreign-born persons with >10 years residence in the United States and no history of subsequent travel, and by Earnest et al. (*19*), who documented 5 such cases with >7 years residence (Table 2). Given that the median incubation period for cysticercosis has been estimated to be 3.5 years (*26*), some of these cases may reflect additional local transmission and thus may indicate a risk for exposure to visitors (family and/or friends) from areas endemic for *T. solium* tapeworm infection or exposure at a social event where visitors or recent immigrants may have helped prepare food. Similarly, 32 cases of cysticercosis in US-born residents who had traveled to disease-endemic areas were noted in the articles reviewed. Given our strict definition, these were considered as travel-related cases but may in fact include cases of authochthonous cysticercosis.

**Table 2:** Reports of cysticercosis in immigrants with long-term residency and no history of foreign travel after arrival in the United States

Location	No. cases	Mean years of residency	Reference
Los Angeles, CA	7	15.4	(*16*)
Denver, CO	5	>7	(*19*)
Los Angeles, CA	20	>10	(*20*)

Among this series of US-acquired cysticercosis a probable source (*T. solium* tapeworm carrier) was identified for 6 cases and presumptive source (close contacts from disease-endemic areas) for 10. This finding represents 21% of the cases despite the fact that information on a source of infection was not reported, and perhaps not sought, for most cases. In the Los Angeles County pilot surveillance system, follow-up, which included examination of stool samples from close contacts, was conducted for 72 patients. A tapeworm carrier(s) was found among contacts for 5 (7%) of these patients. Although the numbers were small, a probable source was identified more commonly among US-born patients (22%) than among those who were foreign born (5%).

## Discussion

Cysticercosis is generally viewed as a disease of developing countries or immigrants from areas where the disease is endemic. However, our review underscores that cysticercosis acquired in the United States can occur in many geographic regions of the country. Moreover, when looked for, a likely source of infection can frequently be found, principally among household members who are major sources of eggs and therefore infection. Like other fecal–oral-transmitted diseases, cysticercosis can be spread either directly or through contaminated food. Persons infected with the adult *T. solium* tapeworms are typically asymptomatic and may not be aware of their infection or of the potential risk to themselves and others. If hygiene is poor, transmission of eggs may occur, particularly within households where repeated opportunities for exposure exist. Even in areas where cysticercosis is endemic, the disease is recognized as a focal disease with clustering of cases identified around tapeworm carriers (*27*). This focal nature makes cysticercosis particularly amenable to public health follow-up and directed control efforts. The ability to find a probable source of infection among contacts to patients with cysticercosis shows that public health follow-up can be successfully conducted. Treatment of tapeworm carriers can eliminate them as possible sources of continuing infection. Such follow-up is routinely conducted by the Los Angeles County Department of Public Health.

The number of cases of cysticercosis acquired in the United States reported in the biomedical literature is clearly a minimum estimate. Because cysticercosis is not a notifiable condition in most jurisdictions and surveillance systems are rarely implemented, reliable information on US transmission is unavailable, and the true prevalence of locally acquired disease is largely unknown. Therefore, it is uncertain what proportion of actually occurring autochthonous cases our review represents. We are aware of several recognized cases of cysticercosis acquired in the United States, including cases in which a source has been determined, but not reported in the literature (M. Tormey, pers. comm.). Moreover, as our review indicates, several publications have documented cysticercosis in immigrants with long-term continuous residence in the United States; some of these infections may have been locally acquired. It is also possible that cysticercosis cases assumed to have been travel related may, in fact, have been autochthonous. In addition, analysis of US mortality data identified 33 cysticercosis deaths among US-born residents over a 13-year period (1990–2002), which represented 15% of all cysticercosis deaths (*28*). However, it was not possible to ascertain from mortality records how many, if any, of these cases may have been acquired in the United States.

Our findings must be viewed with caution. Given that more than half of the case-patients were from Los Angeles County, where travel back and forth to Mexico may be more frequent than it is elsewhere, our findings may be skewed and possibly overestimate the probability of finding a source of infection. In addition, it is uncertain how many studies involved chart review versus patient interview for determining history of exposure in a disease-endemic area, and it is possible some cases reported as locally acquired may have been imported.

Emigration from taeniasis/cysticercosis-endemic areas to the United States is common. In 2008, ≈3.4 million immigrants from Mexico, >700,000 from Central and South American countries, and >1 million from areas of Asia were legal permanent residents of the United States (*29*). Moreover, undocumented immigration from such areas continues to occur in considerable numbers. The US Immigration and Naturalization Service estimates that 11.8 million unauthorized immigrants, nearly 7 million of them from Mexico, resided in the United States in January 2007, and an average of 470,000 persons emigrate from foreign countries each year (*30*). Cysticercosis and taeniasis are widely prevalent in Latin America. Although data are limited, a high prevalence of tapeworm carriers has been observed in these populations. In a study of migrant farmworkers in southern California, DeGiorgio et al., using a sensitive and specific serologic test developed by the Centers for Disease Control and Prevention (Atlanta, GA, USA), documented a *T. solium* tapeworm prevalence of 1.1% (*31*). This level is comparable to that observed in disease-endemic areas. A survey of intestinal parasites among farmworkers in North Carolina found that 3% of workers from Central American countries had *Taenia* spp. tapeworm eggs in their stools (*32*). Because *T. solium* tapeworm eggs are morphologically indistinguishable from *T. saginata* tapeworm eggs (beef tapeworm), it was not possible to determine how many of these represented *T. solium* tapeworm infection; however, *T. saginata* tapeworms, which do not cause human cysticercosis, are less common than *T. solium* tapeworms in Central America. Cardenas et al. reported finding *Taenia* spp. tapeworm in 3.3% of selected residents tested from the Ciudad Juarez, Mexico, and El Paso, Texas, border communities (*33*).

Cysticercosis infection acquired in the United States may also occur through consumption of food contaminated by a *T. solium* tapeworm*–*infected commercial food handler or, theoretically, from contaminated produce. Under favorable conditions, *Taenia* spp. tapeworm eggs can survive for relatively long periods in the environment (*34*), and human feces used as fertilizer or contaminated water used for irrigation can contaminate crops before importation. Transmission of several infectious agents, including hepatitis A (linked to imported green onions from Mexico) and *Cyclospora cayatenensis* (associated with raspberries imported from Guatemala), has been reported (*35**,**36*). Although the report must be viewed with caution, *Taenia* spp. tapeworm eggs were recovered from several varieties of vegetables obtained in local markets in the northeastern Mexican state of Tamaulipas (which borders the United States), suggesting the possibility of transmission from contaminated produce (*37*). Whether these eggs represented *T. solium* tapeworms and were viable is unknown, and this finding has not been verified by other studies. US transmission of cysticercosis linked to contaminated food products has never been documented, and the small number of reported cases of autochthonous cysticercosis may indicate that the risk is low. However, underreporting of the disease and its long incubation period make study of this possible phenomenon difficult.

Several aspects of cysticercosis provide compelling rationales for implementation of public health efforts for the control of this disease. Cysticercosis is a preventable severe infection that can include long-term neurologic sequelae and death. Moreover, it is a fecal–oral-transmitted disease, and a probable source of infection among contacts can frequently be found. In addition, a sensitive (95%) and specific (100%) immunoblot serologic test that uses adult worm antigens is available for identification of tapeworm carriers; however, the duration of antibody response is unknown and could therefore reduce the specificity of identifying active infection in field use (*38*). Nonetheless, this test can be performed on a blood specimen obtained from a finger stick and is more sensitive than stool examination for diagnosis. In addition, stool specimens may be difficult to obtain, multiple specimens are recommended, a skilled microscopist is required, and *Taenia* spp. tapeworms cannot be differentiated from each other on the basis of egg morphologic appearance. Given these factors, the availability of this serologic test substantially improves the ease and potential for follow-up. Adding to the rationale for public health action is the recognition that the economic effect of cysticercosis is considerable. A review of hospital discharge data in Los Angeles County estimated hospitalization costs >$100 million for a 17-year period (*39*).

We believe there is strong justification for routine public health response to a case of cysticercosis. Such a response should include establishing surveillance for the disease and required reporting of cases. When cases are identified, follow-up and testing of household members and other close contacts should be initiated in an attempt to find tapeworm carriers. Such carriers can then be treated and removed as sources of continuing transmission. Investigation of locally acquired cysticercosis cases should be standard public health practice. Although data are limited, tapeworm carriers can also be found among contacts to foreign-born patients (≈5% of the time), and therefore investigation should be considered for all cysticercosis cases; however, imported cases with inactive infection (calcified lesions, indicating probable remote infection) should be a low priority for such follow-up (*16*). Decisions of prioritizing surveillance and control activities must be made on the basis of existing resources and competing needs. Given that a substantial (>20%) proportion of persons with cysticercosis may also be infected with the adult tapeworm, it is also advisable to screen cysticercosis patients if the diagnosing physician has not performed this screening (*26*). Public health authorities should also be aware that a single tapeworm carrier may be a source of infection for multiple cases of cysticercosis; therefore, the possibility of a common exposure among cases should be evaluated. As part of the public health control efforts for cysticercosis, any *Taenia* spp. tapeworm carriers who work as food handlers should be removed from work until successfully treated or confirmation is obtained that the infection is not *T. solium* tapeworm. Screening of domestic workers from areas where the disease is endemic has also been recommended (*3*).

Consideration should be given to making cysticercosis a nationally notifiable disease. Currently, only 2 states, California and Oregon, require reporting of cysticercosis. In collaboration with the Centers for Disease Control and Prevention, the Department of Public Health in Los Angeles County is conducting modified active surveillance that includes education of local providers and routine contact with key health care facilities that treat large numbers of cysticercosis cases. When a case is reported, public health nurses initiate an investigation and follow-up that includes obtaining a finger-stick specimen for serologic testing on all close contacts to identify a possible source of infection. When found, persons harboring a *T. solium* tapeworm are treated to prevent possible ongoing transmission. With heightened awareness, improved surveillance, reporting, and follow-up, cysticercosis transmission in the United States can be prevented and the infection’s effects on public health can be reduced.

## References

[1] Schantz PM, Wilkins PP, Tsang VC. Immigrants, imaging, and immunoblots: the emergence of neurocysticercosis as a significant public health problem. In: Scheld WM, Craig WA, Hughes JM, editors. Emerging infections. Washington: ASM Press; 1998. p. 213–42.

[2] Garcia HH, Del Brutto OH; Cysticercosis Working Group in Peru. Neurocysticercosis: updated concepts about an old disease. Lancet Neurol. 2005;4:653–61. 10.1016/S1474-4422(05)70194-016168934

[3] Phiri IK, Ngowi H, Afonso S, Matenga E, Boa M, Mukaratirwa S, The emergence of *Taenia solium* cysticercosis in eastern and southern Africa as a serious agricultural problem and public health risk. Acta Trop. 2003;87:13–23.1278137410.1016/s0001-706x(03)00051-2

[4] Schantz PM, Wikins PP, Tsang VC. *Taenia solium* cysticercosis as an imported disease. In: Garcia HH, Martinez SM, eds. *Taenia solium*, taeniasis/cysticercosis Lima, Peru: Editorial Universo; 1999. p. 263–72.

[5] Richards FO, Schantz PM, Ruiz-Tiben E, Sorvillo FJ. Cysticerosis in Los Angeles County. JAMA. 1985;254:3444–8. 10.1001/jama.254.24.34444068185

[6] Hotez PJ. Neglected infections of poverty in the United States of America. PLoS Negl Trop Dis. 2008;2:e256. 10.1371/journal.pntd.000025618575621PMC2430531

[7] Wallin MT, Kurtzke JF. Neurocysticerosis in the United States: review of an important emerging infection. Neurology. 2004;63:1559–64.1553423610.1212/01.wnl.0000142979.98182.ff

[8] Schantz PM, Moore AC, Munoz JL, Hartman BJ, Schaefer JA, Aron AM, Neurocysticercosis in an Orthodox Jewish community in New York City. N Engl J Med. 1992;327:692–5. 10.1056/NEJM1992090332710041495521

[9] Mody R, Nield LS, Stauffer W, Kamat D. Seizures in a 20-month-old native of Minnesota. Pediatr Emerg Care. 2005;21:860–2. 10.1097/01.pec.0000190232.20233.4516340766

[10] Stamos JK, Rowley AH, Hahn YS, Chadwick EG, Schabtz PM, Wilson M. Neurocysticercosis: report of unusual pediatric cases. Pediatrics. 1996;98:974–7.8909499

[11] Townes JM, Hoffman CJ, Kohn MA. Neurocysticerccosis in Oregon, 1995–2000. Emerg Infect Dis. 2004;10:508–10.1510942410.3201/eid1003.030542PMC3322801

[12] Rosenfeld EA, Byrd SE, Shulman ST. Neurocysticercosis among children in Chicago. Clin Infect Dis. 1996;23:262–8. 10.1093/clinids/23.2.2628842261

[13] Shandera WX, White AC, Chen JC, Diaz P, Armstrong R. Neurocysticercosis in Houston, Texas: a report of 112 cases. Medicine (Baltimore). 1994;73:37–52.830936110.1097/00005792-199401000-00004

[14] Centers for Disease Control and Prevention. Locally acquired neurocysticercosis—North Carolina, Massachusetts, and South Carolina, 1989–1991. . MMWR Morb Mortal Wkly Rep. 1992;41:1–4.1727973

[15] Kruskal BA, Moths L, Teele DW. Neurocysticercosis in a child with no history of travel outside the continental United States. Clin Infect Dis. 1993;16:290–2. 10.1093/clind/16.2.2908443310

[16] Sorvillo FJ, Waterman SH, Richards FO, Schantz PM. Cysticercosis surveillance: locally acquired and travel-related infections and detection of intestinal tapeworm carriers in Los Angeles County. Am J Trop Med Hyg. 1992;47:365–71.152415010.4269/ajtmh.1992.47.365

[17] Grossman EA. Cysticercosis acquired in the United States, without compatible travel history. Neurology. 1986;36:305.394540810.1212/wnl.36.2.305-a

[18] Mitchell WG, Crawford TO. Intraparenchymal cerebral cysticercosis in children: diagnosis and treatment. Pediatrics. 1988;82:76–82.3288959

[19] Earnest MP, Reller LB, Filllely CM, Grek AJ. Neurocysticercosis in the United States: 35 cases and a review. Rev Infect Dis. 1987;9:961–79. 10.1093/clinids/9.5.9613317739

[20] McCormick GF. Cysticercosis—review of 230 patients. Bull Clin Neurosci. 1985;50:76–101.3842090

[21] Sorvillo FJ, Brecht C, Chambers P, Waterman SH. Cysticercosis transmission in a non-endemic area. Journal of Border Health. 1988;4:2–5.

[22] Tasker WG, Plotkin SA. Cerebral cysticercosis. Pediatrics. 1979;63:761–3.440898

[23] Messner KH, Kammerer WS. Intraocular cysticercosis. Arch Ophthalmol. 1979;97:1103–5.44414310.1001/archopht.1979.01020010557010

[24] Campana M, Swartzwelder C, Comer O. Human cysticercosis in the United States. J Parasitol. 1954;40(Oct Suppl):46.

[25] Dent JH. Cysticercosis cerebri–cestode infestation of human brain; report of a case occurring in Louisiana. J Am Med Assoc. 1957;164:401–5.1341599710.1001/jama.1957.62980040001011

[26] Dixon HB, Lipscomb FM. Cysticercosis: an analysis and follow-up of 450 cases. Spec Rep Ser Med Res Counc (G B). 1961;299:1–58.

[27] Lescano AG, Garcia HH, Gilman RH, Gavida CM, Tsang VC, Rodriguez S, *Taenia solium* cysticercosis hotspots surrounding tapeworm carriers: clustering on seroprevalence but not on seizures. PLoS Negl Trop Dis. 2009;3:e371. 10.1371/journal.pntd.000037119172178PMC2625436

[28] Sorvillo FJ, DeGiorgio C, Waterman SH. Deaths from cysticercosis in the United States. Emerg Infect Dis. 2007;13:230–5. 10.3201/eid1302.06052717479884PMC2725874

[29] Department of Homeland Security. Washington: estimates of the legal permanent resident population in 2008; updated 2009 October [cited 2009 Dec 2]. http://www.dhs.gov/files/statistics/immigration.shtm

[30] Department of Homeland Security. Washington: estimates of the unauthorized immigrant population residing in the United States: January 2007 [cited 2010 Oct 26]. http://www.dhs.gov/xlibrary/assets/statistics/publications/ois_ill_pe_2007.pdf

[31] DeGiorgio C, Pietsch-Escueta S, Tsang V, Corral-Leyva G, Ng L, Medina MT, Sero-prevalence of *Taenia solium* cysticercosis and *Taenia solium* taeniasis in California, USA. Acta Neurol Scand. 2005;111:84–8. 10.1111/j.1600-0404.2005.00373.x15644066

[32] Ciesielski SD, Seed JR, Oriz JC, Metts J. Intestinal parasites among North Carolina migrant farmworkers. Am J Public Health. 1992;82:1258–62. 10.2105/AJPH.82.9.12581503168PMC1694334

[33] Cardenas VM, Mena KD, Otiz M, Karri S, Variyam E, Behravesh CB, Hyperendemic *H. pylori* and tapeworm infections in a U.S.–Mexico border population. Public Health Rep. 2010;125:441–7.2043303910.1177/003335491012500313PMC2848269

[34] Gemmell MA. Taeniidae: modification to the lifespan of the egg and the regulation of tapeworm populations. Exp Parasitol. 1977;41:314–28. 10.1016/0014-4894(77)90104-7849765

[35] Wheeler C, Vogt TM, Armstrong GL, Vaughan G, Weltman A, Nainan OV, An outbreak of hepatitis A associated with green onions. N Engl J Med. 2005;353:890–7. 10.1056/NEJMoa05085516135833

[36] Herwaldt BL, Ackers ML; The Cyclospora Working Group. An outbreak in 1996 of cyclosporiasis associated with imported raspberries. N Engl J Med. 1997;336:1548–56. 10.1056/NEJM1997052933622029164810

[37] Rivero LB, Navarro A. Prevalence of *Taenia solium* eggs in vegetables for human consumption in Cd. Victoria, Tamaulipas. Border Epidemiological Bulletin. 1986;14:1–4.

[38] Wilkins PP, Allan JC, Verastegui M, Acosta M, Eason AG, Garcia HH, Development of a serologic assay to detect *Taenia solium* taeniasis. Am J Trop Med Hyg. 1999;60:199–204.1007213610.4269/ajtmh.1999.60.199

[39] Croker C, Reporter R, Mascola L. Use of statewide hospital discharge data to evaluate the economic burden of neurocysticerocis in Los Angeles County (1991–2008). Am J Trop Med Hyg. 2010;83:106–10. 10.4269/ajtmh.2010.09-049420595487PMC2912585

# Review article: Public Health Implications of Cysticercosis Acquired in the United States

**DOI:** 10.3201/eid1701.101210

**Authors:** Frank Sorvillo, Patricia Wilkins, Shira Shafir, Mark Eberhard

**Affiliations:** Author affiliations: University of California, Los Angeles, California, USA (F. Sorvillo, S. Shafir);; Touro University International, Cypress, California, USA (F. Sorvillo);; Los Angeles County Department of Public Health, Los Angeles (F. Sorvillo);; Centers for Disease Control and Prevention, Atlanta, Georgia, USA (P. Wilkins, M. Eberhard)

## Earning CME Credit

To obtain credit, you should first read the journal article. After reading the article, you should be able to answer the following, related, multiple-choice questions. To complete the questions and earn continuing medical education (CME) credit, please go to www.medscapecme.com/journal/eid. Credit cannot be obtained for tests completed on paper, although you may use the worksheet below to keep a record of your answers. You must be a registered user on Medscape.com. If you are not registered on Medscape.com, please click on the New Users: Free Registration link on the left hand side of the website to register. Only one answer is correct for each question. Once you successfully answer all post-test questions you will be able to view and/or print your certificate. For questions regarding the content of this activity, contact the accredited provider, CME@medscape.net. For technical assistance, contact CME@webmd.net. American Medical Association’s Physician’s Recognition Award (AMA PRA) credits are accepted in the US as evidence of participation in CME activities. For further information on this award, please refer to http://www.ama-assn.org/ama/pub/category/2922.html. The AMA has determined that physicians not licensed in the US who participate in this CME activity are eligible for *AMA PRA Category 1 Credits*™. Through agreements that the AMA has made with agencies in some countries, AMA PRA credit is acceptable as evidence of participation in CME activities. If you are not licensed in the US and want to obtain an AMA PRA CME credit, please complete the questions online, print the certificate and present it to your national medical association.

## Article Title: Public Health Implications of Cysticercosis Acquired in the United States

## CME Questions

**1. Which of the following statements about the presentation and prevalence of cysticercosis acquired within the United States is correct?**A. Cysticercosis primarily presents as pulmonary diseaseB. In this report, cysticercosis was detected in all 50 US statesC. Cysticercosis was primarily reported in immigrants from AsiaD. Cysticercosis is a severe, potentially fatal disease posing a considerable economic burden**2. Your patient is a 27-year-old woman whose parents are immigrants from Latin America. She presents with seizures and is thought to have neurocysticercosis.Which of the following statements about transmission of this illness is most likely correct?**A. Droplet inhalation is the presumed route of infectionB. The responsible pathogen is a hookwormC. A presumptive source of infection may be identified among household members or close personal contacts in about 20% of casesD. There is no good serologic test to confirm the diagnosis3. The patient has positive serology for i and is diagnosed with neurocysticercosis, as is her brother. Which of the following public health strategies would most likely be recommended?A. Examination of urine samples from close contactsB. Only persons with recent travel to endemic areas should be examinedC. A source of infection should be looked forD. Carriers do not need to be treated

## Activity Evaluation

**Table:** 

1. The activity supported the learning objectives.				
Strongly Disagree				Strongly Agree
1	2	3	4	5
2. The material was organized clearly for learning to occur.				
Strongly Disagree				Strongly Agree
1	2	3	4	5
3. The content learned from this activity will impact my practice.				
Strongly Disagree				Strongly Agree
1	2	3	4	5
4. The activity was presented objectively and free of commercial bias.				
Strongly Disagree				Strongly Agree
1	2	3	4	5
